# Unveiling the antimicrobial, antivirulence, and wound-healing accelerating potentials of resveratrol against carbapenem-resistant *Pseudomonas aeruginosa* (CRPA)-septic wound in a murine model

**DOI:** 10.1007/s10787-024-01591-z

**Published:** 2024-11-07

**Authors:** Rana Elshimy, Riham A. El-Shiekh, Mona M. Okba, Rehab M. S. Ashour, Marwa A. Ibrahim, Eman I. Hassanen, Hassan Aboul-Ella, Merhan E. Ali

**Affiliations:** 1 Department of Microbiology and Immunology, Faculty of Pharmacy, Ahram Canadian University, Giza, Egypt; 2 Department of Microbiology and Immunology, Egyptian Drug Authority, Cairo, Egypt; 3 Department of Pharmacognosy, Faculty of Pharmacy, Cairo University, Giza, Egypt; 4 Department of Biochemistry and Molecular Biology, Faculty of Veterinary Medicine, Cairo University, Giza, Egypt; 5 Department of Pathology, Faculty of Veterinary Medicine, Cairo University, Giza, Egypt; 6 Department of Microbiology, Faculty of Veterinary Medicine, Cairo University, Giza, Egypt

**Keywords:** Carbapenem-resistant *P. aeruginosa*, Resveratrol, Gene expression, Murine model, Stalled wound

## Abstract

*Pseudomonas aeruginosa* is a repertoire of several virulence factors that create a frightening high pathogenicity level as well as high antimicrobial resistance toward commercially used antibiotics. Therefore, finding a new alternative to traditional antimicrobials is a must. Resveratrol is a very famous phytochemical that harbors many beneficial health properties by possessing antibacterial, anti-inflammatory, and antioxidant properties. The current study aimed to explore the antimicrobial efficacy of resveratrol against* P. aeruginosa* and explore its ability to accelerate wound healing in a murine model. The obtained results revealed the potent antimicrobial, antivirulence, and wound-healing accelerating potentials of resveratrol against carbapenem-resistant *P. aeruginosa* (CRPA)-septic wounds. It significantly lowered the transcript levels of *P. aeruginosa* virulent genes *tox*A, *pel*A, and *las*B. Additionally, resveratrol significantly accelerated skin wound healing by shortening the inflammatory phase and promoting re-vascularization, cell proliferation, re-epithelialization, and collagen deposition. Furthermore, it increased the immunoexpression of *αSMA* along with a reduction of the mRNA levels of VEGF, *IL-1β,* and *TNF-α* genes. Resveratrol has high therapeutic potential for the treatment of *P. aeruginosa* wound infection and is a prospective and promising candidate for this problem.

## Introduction

*Pseudomonas aeruginosa *(*P. aeruginosa*) is a common opportunistic human pathogen. It often causes various complicated acute and chronic infections in both immunocompetent and immunocompromised hosts. *P. aeruginosa* can multiply and become the main pathogen in patients with cystic fibrosis (CF), ventilator-associated pneumonia, urinary tract infection, otitis externa, burn and wound injuries, bone and joint infections, and systemic infections. Antimicrobial resistance (AMR) is a serious concern to public health around the world. It has been linked to about 5 million fatalities in 2019 and at least 1.27 million deaths globally (Ranjbar and Alam 2024). The 2019 Antibiotic Resistance Threats Report from the Centers for Disease Control and Prevention (CDC) highlights 2.8 million illnesses and 35,000 deaths annually in the USA alone due to antibiotic resistance. Serious nosocomial infections are frequently caused by *P. aeruginosa* isolates that are carbapenem resistant, multidrug resistant (MDR), extensively drug resistant (XDR), and pan-drug resistant (PDR). These isolates are also important sources of morbidity and mortality.

In 2021, 8.9% of *P. aeruginosa* isolates were MDR, based on CDC data in 2021. According to a thorough National Healthcare Safety Network report, patients in intensive care units accounted for 18.6% of MDR isolates, long-term acute-care hospitals for 29.9%, and hospital cancer units for 11.6% (Weiner-Lastinger et al. 2020). One of the six MDR ESKAPE pathogens that causes potentially fatal nosocomial infections is *P. aeruginosa*. The other pathogens are *Enterococcus faecium, Staphylococcus aureus, Klebsiella pneumoniae, Acinetobacter baumannii,* and *Enterobacter *spp. The World Health Organization (WHO) designated carbapenem-resistant *P. aeruginosa* as a critical-priority pathogen in 2017. This means that to combat the escalating global public health issue, new antimicrobials must be developed immediately (Tacconelli et al. 2018). In January 2023, CDC reported an outbreak of a rare strain of XDR *P. aeruginosa* linked to eye drops. As of 15 May 2023, 81 patients were identified in the USA as part of the outbreak (Grossman et al. 2024).

Many different types of wounds are susceptible to bacterial infections. Acute wounds, which include cuts, burns, surgical incisions, and traumatic injuries, are frequently caused by an exterior breach of the patient’s skin (Bowler et al. 2001). On the other hand, long-term disruptions to the patient’s skin barrier function result in chronic wounds. These disruptions are usually caused by comorbidities like diabetes and peripheral vascular disease, which impair the preservation and repair of dermal tissue. Due to the dearth of available treatments, *P. aeruginosa* is one of the most well-known Gram-negative aerobes that causes persistent wound infections (Breidenstein et al. 2011; Pang et al. 2019). The importance of identifying *P. aeruginosa* from a patient's infected wound is still debatable, even though it is linked to persistent wound infections (Esposito et al. 2008). While some medical professionals believe *P. aeruginosa* can colonize the wound space and its eradication is not necessary for a chronic wound to heal, another theory contends that *P. aeruginosa* can cause significant tissue damage and should be treated with targeted antibiotics. Many studies have found that *P. aeruginosa* virulence factors affect a wound's capacity to heal, lending credence to the latter theory (Bjarnsholt et al. 2008; Goldufsky et al. 2015; Chen et al. 2018; Prasad et al. 2020).

The most appropriate strategy of action for treating pseudomonal wound infections is further complicated by the uncertainty surrounding the interpretation of clinical microbiology cultures acquired from wound swabs, as well as the challenge of establishing empirically whether *P. aeruginosa* is present in a wound infection*. P. aeruginosa* has been positively associated with wound infections in high-severity infections, lower extremity exposure to water, and living in a warmer climate (Noor et al. 2015; Ghotaslou et al. 2018; Pitocco et al. 2019). Furthermore, the possibility of encountering *P. aeruginosa* in wound infections has been weakly correlated with global geography, with the Eastern Hemisphere, or Asia and Africa in particular, being recognized as having a high frequency of *P. aeruginosa* (Noor et al. 2015; Saeed et al. 2020).

Several plant extracts and pure compounds showed very potent potential against various diseases (Abdel-Baki et al. 2023; Ali et al. 2024a, b; El-Shiekh et al. 2024a; El-Shiekh et al. 2024b; Ghanem et al. 2024; Mahdally et al. 2024). Resveratrol is a well-known polyphenolic secondary metabolite detected in and isolated from various plants (Patel et al. 2011). It has well-documented several health benefits and exhibits significant therapeutic properties. It revealed significant inhibitory potential against various pathogens (*Campylobacter*, *Listeria*, *E. coli*, and *Staphylococcus aureus*) (Zhang et al. 2021). The rising incidence of MDR strains has rendered it extremely important to civilian physicians and military medicine. Novel pharmaceuticals and therapy options for wound infections require urgent research. In vivo testing in animal models is a vital stage in the bench-to-bedside strategy and is required for Food and Drug Administration approval during the drug development and therapeutic testing process. To our knowledge, few data have been published regarding the topical application of resveratrol. Therefore, the current work investigates the antimicrobial, antivirulence, and wound-healing accelerating potentials of resveratrol against a hypervirulent multidrug-resistant carbapenem-resistant *P. aeruginosa* (hvCRPA)-septic wound in a murine model.

## Materials and methods

### Pre-treatment preparatory stage

#### Phenotypic identification and characterization of the obtained strains

The selection of the *P. aeruginosa* strain used during the current study was based on a screening process of several clinical isolates and a *P. aeruginosa* ATCC strain used as a positive control. Therefore, *P. aeruginosa* ATCC 27853 and six clinical isolates (PA1–PA6) were obtained from the wound exudate of tertiary hospital intensive care unit patients in Cairo, Egypt. The *P. aeruginosa* isolate was phenotypically identified using standard microbiological techniques: Gram staining, Cetrimide Agar culturing, and oxidase testing followed by antimicrobial resistance profiling, molecular identification, and characterization.

#### Antimicrobial resistance profiling of the obtained strains

The diffusion method was performed to determine the antibiotic susceptibility profile of the obtained *P. aeruginosa* isolates to commonly used antimicrobials to treat animal and human diseases, including septic wound cases worldwide (Malaka et al. 2018; Boongapim et al. 2021). The results obtained were analyzed and interpreted following the guidelines of the Clinical and Laboratory Standards Institute (CLSI 2024). A total of six different antibiotics (HIMEDIA, India), belonging to five different antibiotic classes, were employed such as cephalosporins (ceftriaxone (CTX)), aminoglycosides (gentamicin (GEN)), monobactams (aztreonam (AZT)), carbapenems (imipenem (IMP) and meropenem (MEP)), and fluoroquinolones (ciprofloxacin (CIP)). Each antimicrobial susceptibility test (AST) was performed two times to confirm and ensure the reproducibility of the results. According to the World Health Organization (WHO), isolates that were resistant to three or more antibiotic classes were considered MDR.

#### Molecular identification and characterization

The molecular identification and characterization were performed on the selected multidrug-resistant carbapenem-resistant *P. aeruginosa* (MDR-CRPA) strain.

#### DNA extraction

DNA extraction was performed using the QIAamp DNA Mini kit (QIAGEN, Germany, GmbH) with minor modifications according to the manufacturer’s recommendations. Oligonucleotide primers were supplied from (METABION, Germany) and are listed in Table 1.

**Table 1:** Primer sequences of *P. aeruginosa* target genes

Target gene	Encoding	Primers sequences	References
*exo*U	Cytotoxin	F: 5′-CCGTTGTGGTGCCGTTGAAG-3′	(Winstanley et al. 2005)
		R: 5′-CCAGATGTTCACCGACTCGC-3′	
*phz*M	Pyocyanin	F: 5′-ATGGAGAGCGGGATCGACAG-3′	(Finnan et al. 2004)
		R: 5′-ATGCGGGTTTCCATCGGCAG-3′	
*tox*A	Exotoxin	F: 5′-GACAACGCCCTCAGCATCACCAGC-3′	(Matar et al. 2002)
		R: 5′-CGCTGGCCCATTCGCTCCAGCGCT-3′	
*las*A	Lactamase A	F: 5′-ATGATCGTACAAATTGGTCGGC-3′	(Bratu et al. 2006)
		R: 5′-GTCATGAAACCGCCAGTCG-3′	
*psl*A	Exoplysaccharides Psl	F: 5′-TCCCTACCTCAGCAGCAAGC-3′	(Ghadaksaz et al. 2015)
		R: 5′-TGTTGTAGCCGTAGCGTTTCTG-3′	
*pel*A	Biofilm matrix	F: 5′-CATACCTTCAGCCATCCGTTCTTC-3′	(Ghadaksaz et al. 2015)
		R: 5′-CGCATTCGCCGCACTCAG-3′	
*las*R	Lactamase R	F: 5′-CTGTGGATGCTCAAGGACTAC-3′	(Saleh et al. 2019)
		R: 5′-AACTGGTCTTGCCGATGG-3′	
*las*B	Lactamase B	F: 5′-ACAGGTAGAACGCACGGTTG-3′	(Finnan et al. 2004)
		R: 5′-GATCGACGTGTCCAAACTCC-3′	
*16S rRNA*	*P. aeruginosa* species-specific primer	F: 5′-GGGGGATCTTCGGACCTCA-3′	(Anuj et al. 2009)
		R: 5′-TCCTTAGAGTGCCCACCCG-3′	

#### PCR amplification conditions

Amplification was performed in a 20 µl reaction volume that consisted of 2 µL 10 × Taq PCR buffer, 1.6 µL of 2.5 mM dNTPs mixture, 1 µL of 10 pmol/ µL of each primer (F and R), 1.5 µL template (20 ng/ µL), and 0.2 µL KOMA-Taq (2.5 U/ µL), and then made up to the final reaction volume (20 µL) using distilled water (HPLC grade). The cycling conditions of the primer sequences during PCR were adjusted as described in previous studies in Table 1.

#### Animals

Twenty 9-week-old healthy male BALB/c mice were used in this experiment. All animals were housed in individual cages under constant temperature (20 ± 2 ℃) with 12 h light/dark cycle and had free access to food and water. Cages were enriched with carton houses, wooden boards, small blocks for gnawing, and wood wool for nesting to facilitate natural behavior before and throughout the experiments.

#### Drugs and phytochemicals

Resveratrol was purchased from Merck, USA. Synonym(s): resveratrol, 3,4′,5-trihydroxy-trans-stilbene, 5-[(1E)-2-(4-hydroxyphenyl) ethenyl]-1,3-benzenediol. Its empirical formula (Hill notation) is C_14_H_12_O_3_ and its CAS number: is 501-36-0.

### Treatment evaluation stage

#### In vitro evaluation of the antimicrobial activity

Resveratrol’s both minimal inhibitory concentration (MIC) and minimal bactericidal concentration (MBC) were evaluated as previously done by Sharififar et al. (2007). Broth microdilution (BMD) assays were performed according to the guidelines of the CLSI. The bacterial solutions were prepared by transferring a single colony into Mueller Hinton broth (MHB, HiMedia, India) and incubated overnight at 37 °C. Antimicrobial concentration ranged from 2 to 1024 μg/mL. Resveratrol was dissolved in dimethyl sulfoxide (DMSO), and the latter was used as a negative control. To determine the MIC, the last well in the series without any visible growth was read. Subcultures were prepared out of each well. The first well in the series without any visible growth indicated the corresponding MBC.

### In vitro evaluation of the antivirulence activity

#### Laboratory incubation of *P. aeruginosa* with ciprofloxacin and resveratrol

*P. aeruginosa* was grown in Luria–Bertani (LB) broth for 18 h, then diluted 1:100 in LB and incubated separately with the minimal inhibitory concentration of resveratrol and ciprofloxacin at 37 °C for 48 h in static conditions. After washing bacterial pellets with Tris–EDTA (TE) buffer, bacterial mRNA was extracted.

#### Molecular assessment of the *P. aeruginosa* virulence genes

RNA extraction was done as previously described (Farahpour et al. 2020). Oligonucleotide primers were supplied from Metabion, Germany and are shown in Table 1. The expression of the virulence gene was analyzed using qRT-PCR and the housekeeping gene 16S rRNA served as an internal control (Goll et al. 2006; Yuan et al. 2006; El-Azzouny et al. 2018).

### In vivo evaluation of the wound-healing effect

**Fig. 1:**
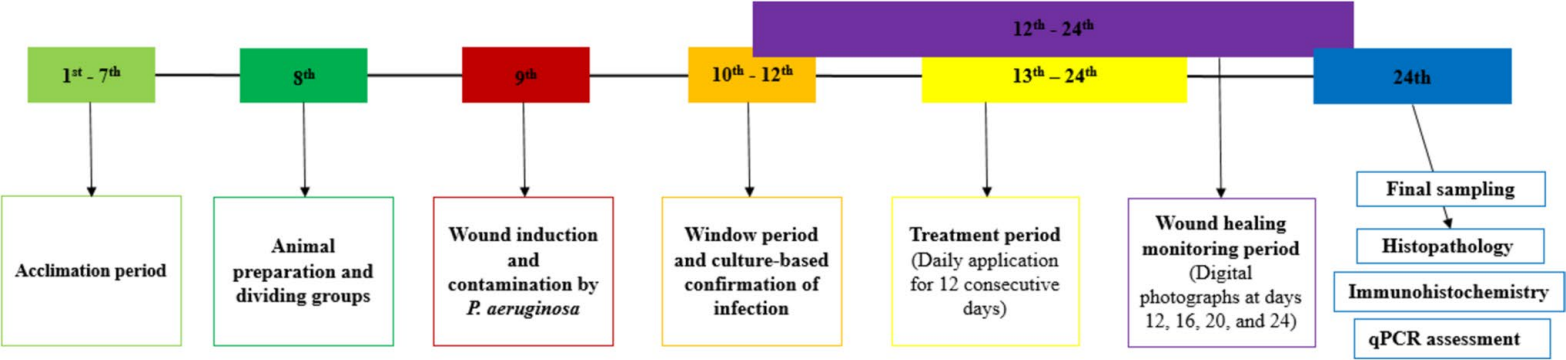
Illustrative diagrammatic flow of the experimental animal stage

The detailed protocol overall flow of the experimental animal stage is illustrated clearly in Fig. 1.

#### Animal acclimation and grouping

Mice were randomly divided into four groups (n = 5): Group 1: non-infected non-treated group (normal saline (placebo) was applied as negative control), Group 2: infected non-treated group (normal saline (placebo) was applied as negative control), Group 3: infected then treated by ciprofloxacin, and Group 4: infected then treated by resveratrol.

#### Wound induction

For developing wounds, mice were anesthetized by injecting a ketamine–xylazine (K–X) cocktail consisting of ketamine (100 mg/kg) (WOERDEN, Holland) and xylazine (10 mg/kg) (Woerden, Holland), via intraperitoneal administration (Levin-Arama et al. 2016). Briefly, the exposed shaved skin area was cleaned with 70% ethanol, and full-thickness skin wounds (5 mm) were created on the dorsal middle line of the mouse. The wounds were left open for the duration of the study without any dressing.

#### Wound contamination and active infection confirmation

Clinical isolate (PA3) was grown in MHB. When bacteria were in the growth log phase, the suspension was centrifuged for 15 min at 1000*g*, then the supernatant was discarded, and the bacteria were diluted in sterile phosphate-buffered saline (PBS) to ~ 10^9^ CFU/mL. Immediately after wound surgery, 25 μL of bacterial suspension was added to each wound bed. Active infection confirmation was done using a basic culturing technique in the following 3 days post-contamination (Hakansson et al. 2014).

#### Treatment application and healing assessment

To be applied on wounds, resveratrol was dissolved in 1% sterile saline and stored at 4 °C for up to 1 month in the dark as indicated by Shevlev et al. (2022). Topical treatments were applied once daily for 12 consecutive days in Carbopol hydrogel on the wound bed: 10 μL of resveratrol in the treated group, and ciprofloxacin in the ciprofloxacin group. Wound healing was monitored both grossly and microbiologically by taking digital photographs and specific plate counts, respectively, on days 0, 4, 8, and 12 post-treatments.

#### Gross lesion monitoring

First, the wound area was checked by measuring the length and width directly with a graduated ruler (Kundin 1989). Second, the relative percentage of tissue types in the wound bed (granulation, slough, and necrotic tissue) was estimated. Third, evaluation of the wound fluids (transudates, exudates, blood, and pus) was done.

#### Bacterial bioburden determination

To determine the bacterial counts in the tissue samples, tissue specimens were individually weighed and homogenized in 2 mL of PBS. The homogenates and the collected wound fluids of each wound were then serially diluted in PBS (1:10, 1:100, 1:1,000, and 1:10,000) and plated on Cetrimide Agar plates in triplicate. Plates were then incubated for at least 18 h at 37 °C under a humidified atmosphere. All colony counts were expressed as log^10^ CFU per milliliter wound (Robson et al. 1999; O'meara et al. 2006).

#### Wound harvesting

Mice were euthanized at the scheduled time point post-wounding following an approved animal protocol. A wide rectangle around the wound sites was cut using a scalpel, and the rectangular piece of tissue was freed using scissors and tweezers to peel back and cut the skin away from the underlying tissue. The harvested wounds were placed in a Petri dish. 2 mm of unwounded tissue surrounding all sides of the wound was trimmed down in a rectangular shape. At least one wound per mouse was used for paraffin embedding and histological analysis (Rhea and Dunnwald 2020).

## Post-treatment assessment stage

### Analysis of the mRNA levels of *TNF-α*, *VEGF*, and *IL-1β*

RNA extraction was performed using the Gene JET RNA purification kit (Thermo Fisher Scientific, USA) (Hassan et al. 2022). The primer sets for the target genes were designed using Primer Premier 5 software, based on the musculus mRNA sequences (Table 2).

**Table 2:** The primer sequences for the target genes

Gene symbol	Gene description	Accession number	Primer sequence
*TNF-α*	Tumor necrosis factor	NM_013693.3	F: 5′- TGTAGCCCACGTCGTAGCAA -3′
			R: 5′- ATAGCAAATCGGCTGACGGT -3′
*IL-1β*	Interleukin 1 beta	NM_008361.4	F: 5′- TGCCACCTTTTGACAGTGATG -3′
			R: 5′- AAGGTCCACGGGAAAGACAC -3′
*VEGF*	Vascular endothelial growth factor A	NM_001025250.3	F: 5’-GGGAGTCTGTGCTCTGGGAT-3’
			R: 5’-GGTGTCTGTCTGTCTGTCCG-3’
*ACTB*	Beta-actin	NM_007393.5	F: 5′- CCACCATGTACCCAGGCATT -3′
			R: 5′- AGGGTGTAAAACGCAGCTCA -3′

### Histopathological evaluation

The formalin-fixed skin specimens were handled by the traditional method using ethanol at concentrations of 70%, 80%, 90%, and 100% for water removal, followed by xylene for purification. After that, the samples were soaked in paraffin wax and a block was cut into 4.5 μm-thick sections that were stained by hematoxylin and eosin (H&E) and examined by a light microscope (Olympus-BX43). Histographies were captured by an Olympus DP21 camera attached with Cell Sens dimension software (Bancroft et al. 2013).

The morphological features of wound-healing processes were blindly scored on a five-point scale based on the degree of re-epithelialization, inflammation, granulation tissue formation, and collagen deposition as explained in Table 3. The median score was taken from at least 35 microscopic fields (5 microscopic fields/section in 7 sections for each group that representing 7 mice) (Simonetti et al. 2008).

**Table 3:** Grading criteria for morphological evaluation of wound healing

Score	Re-epithelialization	Inflammation	Granulation tissue formation	Collagen deposition
0	None	None	None	None
1	Single layer	Traces (< 25%)	Less cellular and less vascular	Traces
2	Partial epithelial covering	Mild (25–50%)	More cellular and less vascular	Mild
3	Stratified and partially keratinized	Moderate (51–75%)	An equal number of cells, vessels, fibers	Moderate
4	Complete and normal	Severe (> 75%)	Less cellular and more fibrous	Marked

### Immunohistochemical staining

Deparaffinized skin tissue sections were dehydrated by graded ethanol and purified by xylene. Then, sections were subjected to antigen retrieval and incubated with *αSMA* primary antibody (ABCAM, Ltd.), followed by the reagent included in avidin–biotin detection kits (Power‐Stain 1.0 Poly HRP DAP Kit; Sakura). All sections were examined by a light microscope (OLYMPUS-BX43) blindly.

### Statistical analysis

SPSS version 18.0 was utilized to analyze the data. For comparison, Dunnett's test was applied to assess the impact of treatments. Differences were significant at *P* < *0.05*. Student’s *t* test was used to compare the two groups in normal data distribution and the Mann–Whitney *U* test for non-normal distribution.

## Results

### Pre-treatment preparatory stage

#### Phenotypic identification and characterization of the obtained strains

All tested strains were phenotypically identified as *P. aeruginosa* and showed the basic phenotypic characteristics of *P. aeruginosa*: Gram-negative short rods by Gram’s technique, pyocyanin exo-pigmented colonies on Cetrimide Agar, and biochemically oxidase positive and alkaline slant represented by red color/alkaline butt represented by red color (K/K) triple sugar iron (TSI) testing.

#### Antimicrobial resistance profiling of the obtained strains

All tested isolates showed a different one or two-drug resistance profile with one isolate (PA3) showing a multidrug-resistant pattern that was resistant to ceftriaxone, gentamicin, aztreonam, imipenem, and meropenem, and sensitive to ciprofloxacin. Based on the antimicrobial resistance results, the isolate (PA3) has been chosen as the MDR-CRPA model through the subsequent stages of the current work.

#### Molecular identification and characterization

Upon screening of the major *P. aeruginosa* virulence genes, the isolate was negative for *las*R and positive for *pel*A*, psl*A*, las*I*, tox*A*, phz*M*, exo*U, and *las*B showing bands at 786, 656, 606, 396, 875, 134, and 1200 bp, respectively. Therefore, this clinical isolate can be identified as a hypervirulent strain (Supplementary Figures S1 and S2).

### Treatment evaluation stage

#### In vitro evaluation of the antimicrobial activity

The results of the BMD of resveratrol against the used *P. aeruginosa* isolate (PA3) were as follows: the MIC result was 256 μg/mL, while the MBC results were 512 μg/mL.

#### In vitro evaluation of the antivirulence activity

Resveratrol effectively reduces the expression of *tox*A and *pel*A by 70% and 45%, respectively. Consequently, the isolated ability to produce carbohydrate-rich biofilm matrix is reduced allowing an enhanced effect for antimicrobials (Table 4).

**Table 4:** Fold changes in the expression of *pel*A, *tox*A, and *las*B genes before and after treatment by resveratrol

	*16S* rDNA	*pel*A		*tox*A		*las*B	
	CT	Fold change	CT	Fold change	CT	Fold change	CT
Resveratrol-untreated isolate (control)	20.27	–	21.34	–	22.85	–	18.26
Resveratrol-treated isolate	20.39	0.33	23.07	0.55	23.82	0.2045	18.51

*CT* cycle threshold

### In vivo evaluation of the wound-healing effect

#### Gross lesion monitoring

**Fig. 2:**
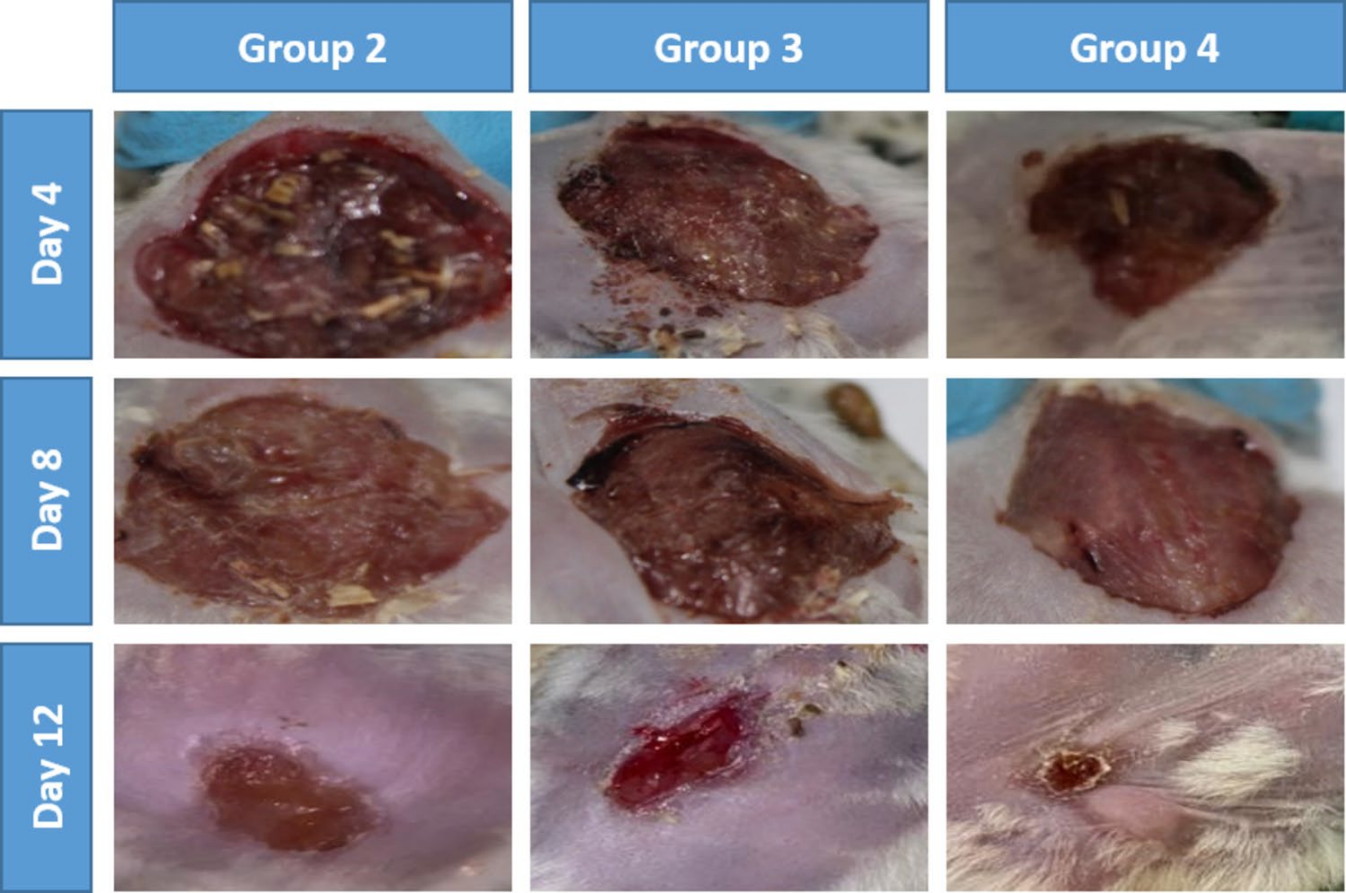
Illustrations of infected full-thickness wound closure in each experimental group on different days after wounding

In the animals treated with ciprofloxacin and resveratrol, the wound area was reduced significantly (*p* < 0.05) compared to the control group following wound induction Fig. 2.

#### Bacterial bioburden determination

Quantification of the bacterial bioburden in each infected wound over time indicates that *P. aeruginosa* load in the ciprofloxacin and resveratrol groups is lower than that in the infected non-treated group. In the present study, topical resveratrol treatment markedly reduced the bacterial load and accelerated the processes of wound healing compared with the ciprofloxacin-treated group Table 5.

**Table 5:** The bacterial bioburden of each animal group over time

	Group 1	Group 2	Group 3	Group 4
Day 0	Undetectable	3.31 × 10^8^ ± 0.577	3.33 × 10^8^ ± 0.3	3.32 × 10^8^ ± 0.25
Day 4	Undetectable	5.6 × 10^10^ ± 0.577	2.5 × 10^6^** ± **1.52	2.3 × 10^6^** ± **1.2
Day 8	Undetectable	5.1 × 10^11^ ± 0.41	2.1 × 10^3^** ± **0.05	2.2 × 10^3^** ± **0.57
Day 12	Undetectable	4.5 × 10^12^ ± 0.5	Uncountable	Undetectable

### Post-treatment assessment stage

#### Analysis of the mRNA levels of *TNF-α, VEGF, *and* IL-1β*

**Fig. 3:**
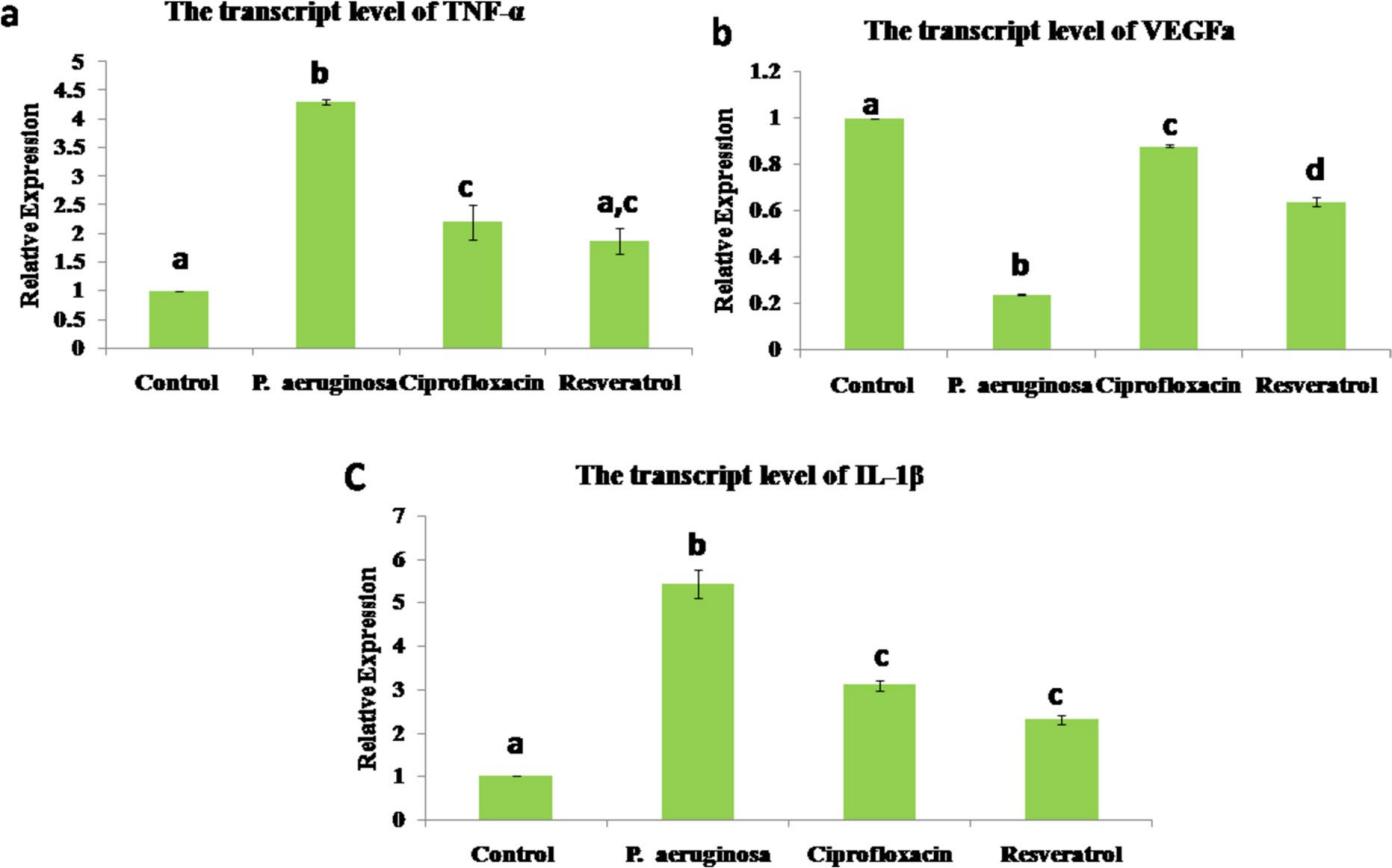
Bar chart representing the transcript levels of a: *TNF-α*, b: *VEGF*, and c: *IL-1β*. Values are presented as mean ± SEM (n = 5 mice/group). Different superscript letters (a, b, c, d) indicate a significant difference at *P* ≤ 0.05

The transcriptase level of *TNF-α* and *IL-1β* recorded a significant upregulation in the *P. aeruginosa*-infected group, whereas *VEGF* showed a significant downregulation in this group. Ciprofloxacin and resveratrol significantly accelerated the repair of infected wounds by downregulating both *TNF-α* and *IL-1β* and upregulating *VEGF* (Fig. 3).

#### Histopathological evaluation

**Fig. 4:**
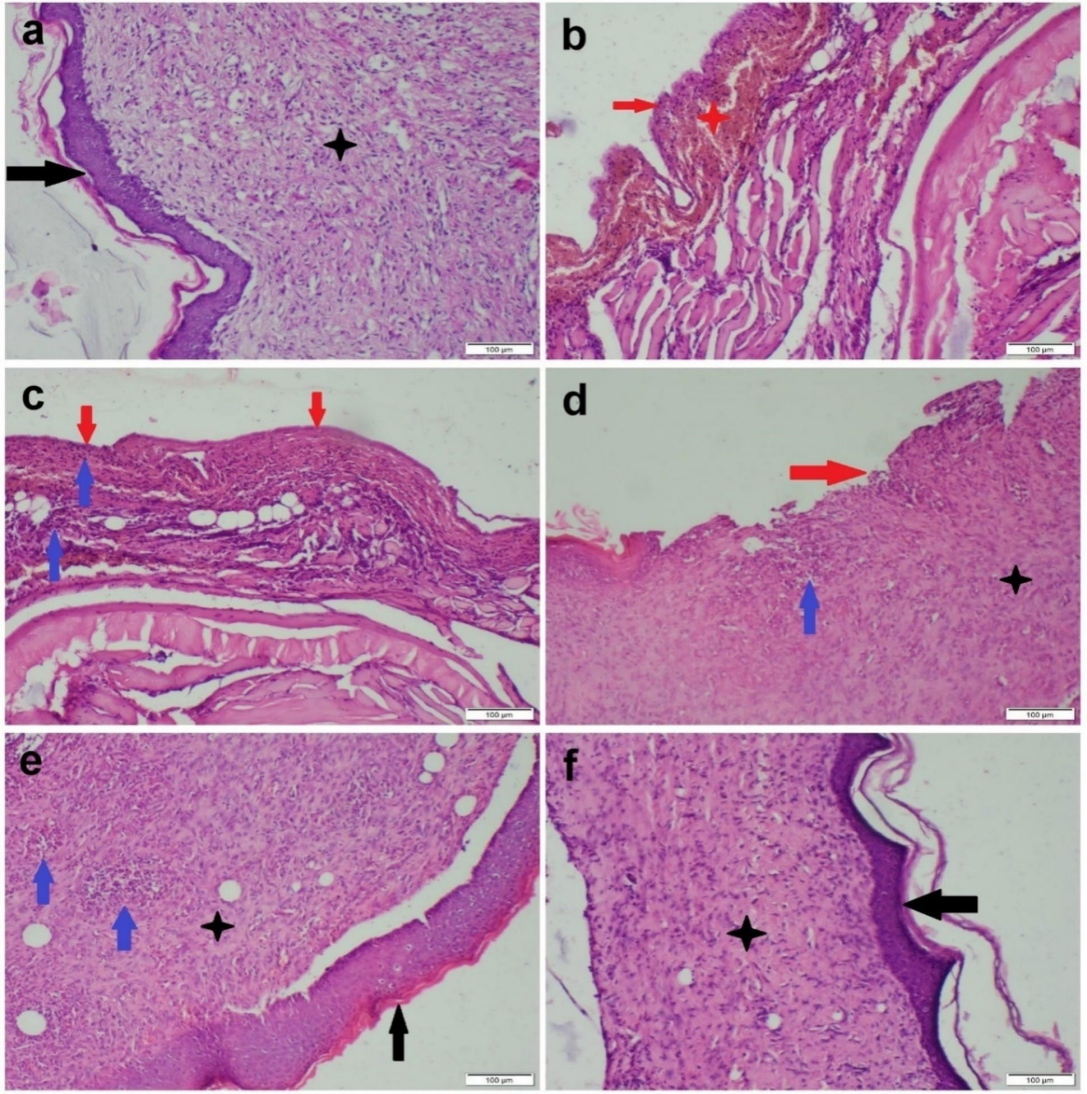
Photomicrographs of H&E-stained skin sections from the wound area of various experimental groups: **a** control non-infected wound showed normal skin repair processes, **b**, **c** *P. aeruginosa*-infected wound showed failure in the processes of wound healing, **d**, **e** ciprofloxacin-treated infected wound showed moderate acceleration of wound-healing procedures, **f** resveratrol-treated infected wound showed marked acceleration of wound-healing procedures. Complete epithelialization (black arrow), well-formed granulation tissue (black star), epidermal ulceration (red arrow), hemorrhage (red star), and pockets of inflammatory cells (blue arrow)

**Fig. 5:**
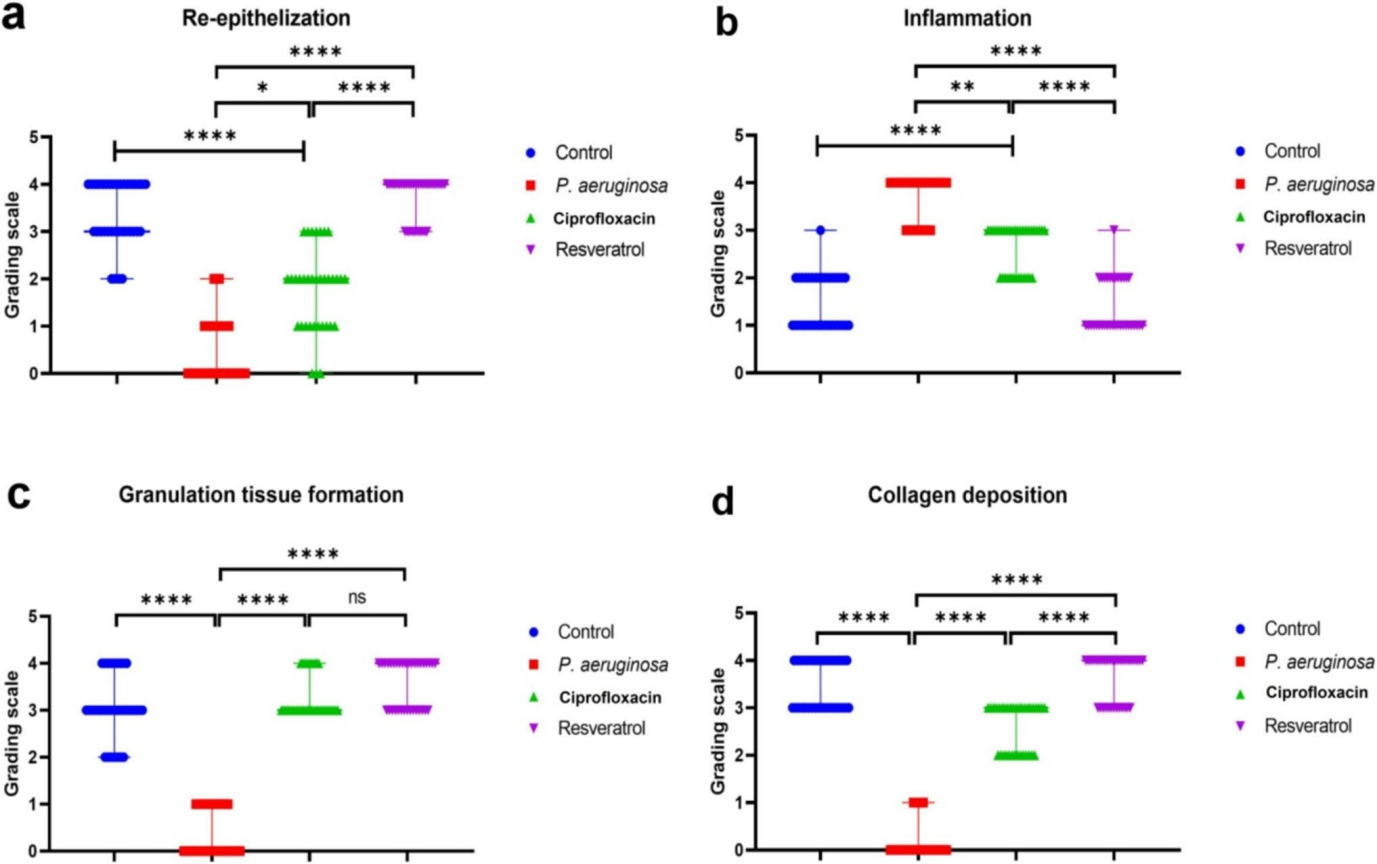
Scatter plot representing the morphological features of wound healing in various groups: **a** re-epithelialization, **b** inflammation, **c** granulation tissue formation, and d collagen deposition. Data presented as median with range (n = 35 microscopic fields/ group), (ns) means non-significant, whereas (⁎), (⁎⁎), and (⁎⁎⁎⁎) indicate significant differences at *P* < *0.05, 0.01,* and *0.0001*, respectively

The control non-infected wound showed normal healing processes manifested by complete re-epithelialization with thick stratified epithelial cells besides granulation tissue formation (Fig. 4a). The granulation tissue contained enormous vascular tissue and well-organized collagen deposition. Otherwise, *P. aeruginosa*-infected wound showed marked retardation in the repair processes. Most sections showed complete epidermal ulceration or were only covered by single epithelial cells. Complete dermal necrosis and hyalinosis were noticed along with extensive hemorrhage and inflammatory cell infiltration Fig. 4b. Some sections showed thin granulation tissue at the wound edge with ill-organized collagen fibers (Fig. 4c). On the other hand, treatment of *P. aeruginosa*-infected wounds with ciprofloxacin moderately accelerated the processes of wound healing compared with *P. aeruginosa*-infected wounds, but still delayed in contrast to the non-infected wound. There was partial re-epithelialization with well-developed granulation tissue in the wound edge and bed (Fig. 4d). The granulation tissue was filled with numerous inflammatory cells, fibroblasts, and exudation besides moderate angiogenesis (Fig. 4e). Resveratrol markedly accelerated the processes of wound healing that demonstrated completely thick stratified epithelial cell covering with well-formed granulation tissue in the wound edge and bed (Fig. 4f). The granulation tissue was less cellular and more fibrous with extensive angiogenesis. Regarding the progression of wound healing in various groups, it is reported that resveratrol-treated group recorded the highest score in the entire healing criteria compared with other groups. Otherwise, the *P. aeruginosa-*infected group had the lowest score in all criteria. Furthermore, the ciprofloxacin-treated group noticed a significant difference in lesion score compared with the untreated wound either infected or non-infected (Fig. 5).

#### Immunohistochemical staining

**Fig. 6:**
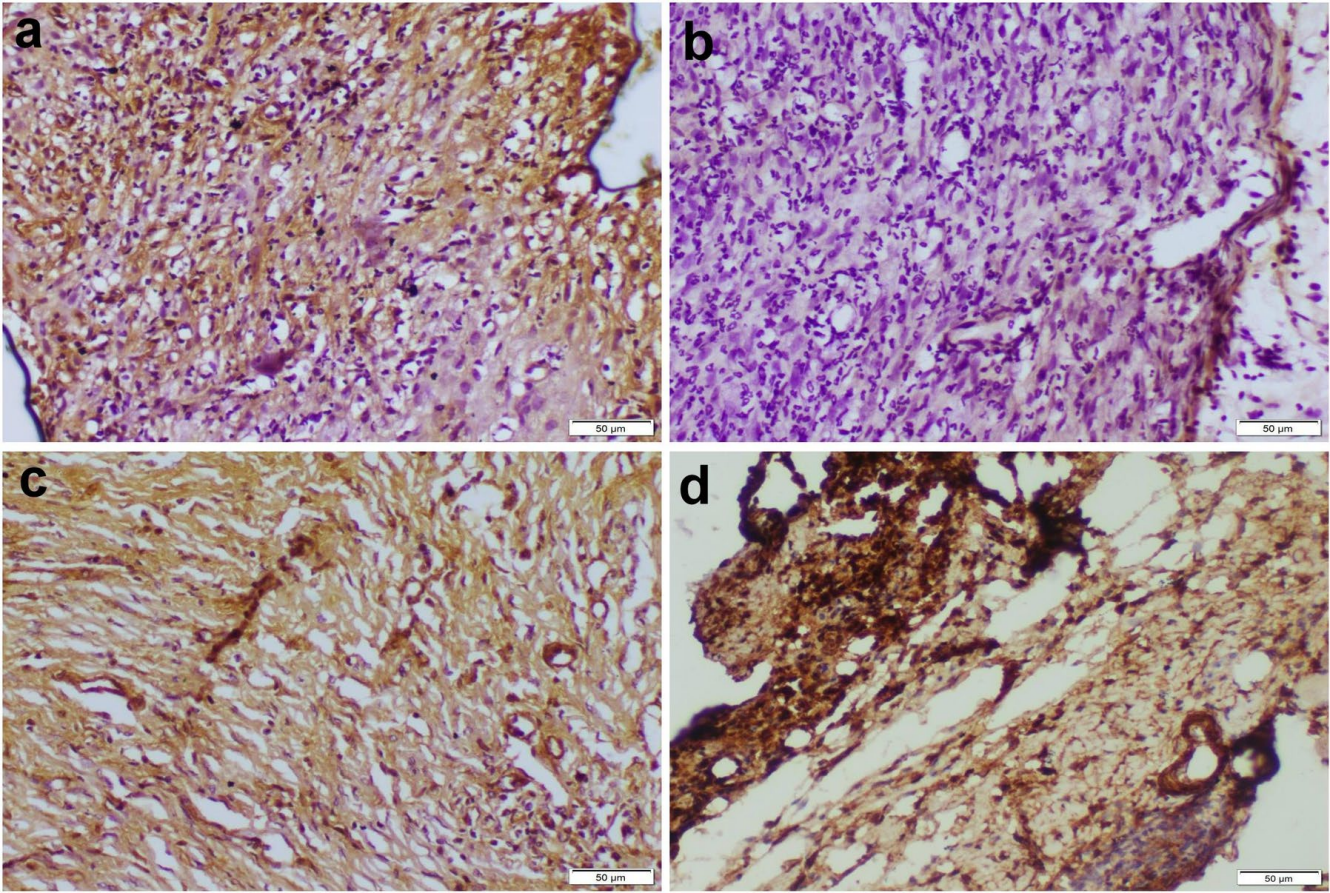
Photomicrographs representing *αSMA* immunostaining within wound area of various experimental groups: **a** control uninfected wound showed moderate *αSMA* immunostaining, **b** *P. aeruginosa*-infected wound showed negative *αSMA* immunostaining, **c** ciprofloxacin-treated infected wound showed moderate *αSMA* immunostaining, and **d** resveratrol-treated infected wound displayed strong *αSMA* immunopositivity

The immunostaining of *αSMA* recorded a weak expression in the *P. aeruginosa*-infected wound, whereas the ciprofloxacin-treated wound showed a moderate *αSMA* similar to those of the control uninfected wound. Resveratrol significantly accelerated the processes of wound healing by increasing the expression of the studied immune marker that exceeded those of the control uninfected wound (Fig. 6).

## Discussion

**Fig. 7:**
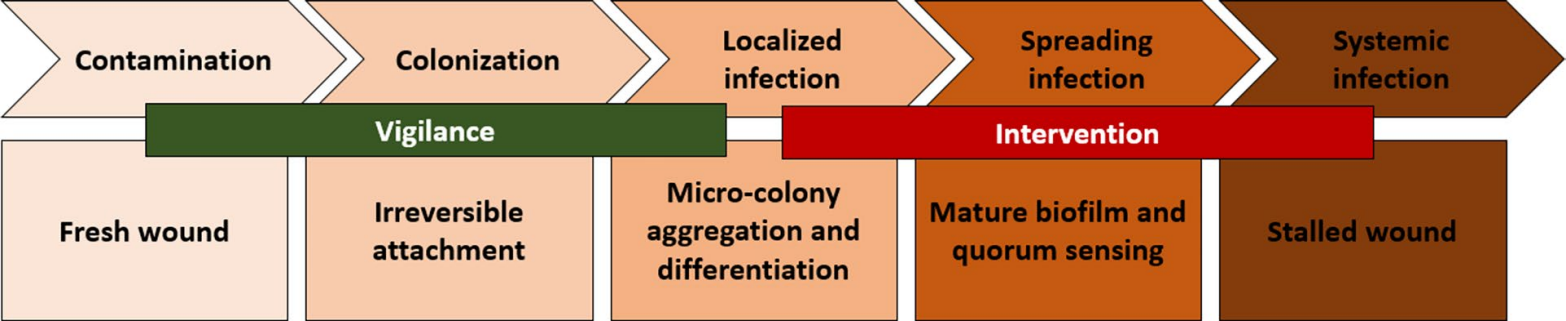
Diagrammatic description of the pathway of a wound from a fresh to stalled stage with illustration of the microbial risk in each stage and the possible time of vigilance and intervention

In recent years, there has been an increasing interest in the healing of wounds. The healing of some mechanical injuries, burns, diabetic wounds, and others has become a clinical challenge, and the resistance to antibiotics in modern medicine has become a therapeutic bottleneck. Contamination of wounds by microbes is considered one of the complicated and worst sequels of the wound-healing process, especially if the contaminating microorganism is one of those microorganisms that have a plenty and wide variable arsenal of virulence and infection-establishing factors (Solanki et al. 2023). The presence of such bacteria in the wound microenvironment will be the beginning spark to interfere with the wound-healing natural and medicinally added process through a multistep process: contamination, colonization, localized infection, spreading infection, and one of the worst dead-end scenarios, systemic infection, which will require an emergent intervention (Fig. 7).

The urgent quest for alternative antimicrobials is imperative due to the escalating issue of antibiotic resistance due to misuse and overuse (Abdel-Sattar and El-Shiekh 2024; El-Shiekh et al. 2024c). Therefore, natural plant extracts, known for their ability to disrupt pathogenic bacterial membranes and contain various beneficial compounds, emerge as potential alternatives. Resveratrol is found in multiple plants, including beans, rice, and grape skins. It has also been found to have anti-inflammatory, antioxidant, antibacterial, and even pro-angiogenic effects in a variety of diseases. Due to the high gastrointestinal degradation and low bioavailability of resveratrol orally, several forms of dressings have been investigated and developed.

Moreover, resveratrol, including its derivatives, possesses potent antimicrobial activity against both Gram-negative and Gram-positive bacteria (Cebrián et al. 2023). Recently, numerous studies have shown that resveratrol has an antibacterial effect on at least 20 kinds of bacteria (Paulo et al. 2010). However, limited research has explored its antibacterial impact on multidrug-resistant carbapenem-resistant *P. aeruginosa* models.

Being one of the most frightening nightmares hindering any successful treatment journey, especially for intensive care unit patients, combating hypervirulent multidrug-resistant *P. aeruginosa* with a suitable, safe antimicrobial has become a priority (Zhen et al. 2019; De Oliveira et al. 2020). *P. aeruginosa* is one of the ESKAPE pathogens whose rising multidrug resistance and virulence include *Staphylococcus aureus, Enterococcus faecium, Klebsiella pneumoniae, P. aeruginosa*, *Acinetobacter baumannii,* and *Enterobacter* spp. (De Oliveira et al. 2020). Those bacteria are the causative agents for major infections and are characterized by “escaping” the antimicrobial agent bactericidal effect (De Oliveira et al. 2020). Regarding *P. aeruginosa*, the high frightening pathogenicity is due to the high virulence capacity that includes various virulence factors such as alkaline protease and elastase, which are associated with tissue destruction of the cornea, lung, and skin during chronic wound infection. Elastase is responsible for necrotic skin lesions that are known as ecthyma gangrenosum (Liao et al. 2022). On the other hand, lipids and lecithin are usually broken down by lipase and lecithinase, resulting in tissue invasion in the infected area. Finally, the extracellular enzyme, exotoxin A, is the true mediator for both local and systemic diseases causing severe dermonecrotic changes. Toxin-positive *P. aeruginosa* isolates, such as the clinical isolate used in this study, had been reported to attain greater virulence and higher *Pseudomonas* septicemia probability compared with toxin-negative strains (Mansor et al. 2023). Furthermore, the *P. aeruginosa* quorum sensing (QS) system significantly promotes cell-to-cell communication enhancing its resistance and hindering any treatment protocol success (Chadha et al. 2022). Resveratrol could efficiently downregulate the gene expression of *tox*A by 70%. Therefore, resveratrol is considered a truly promising agent to overcome severe pseudomonal infected cases because *tox*A is associated with severe chronic infection cases more than pulmonary infection cases, showing high transcription levels of *tox*A. Upon performing MIC, resveratrol showed results ranging from 256 to 512 μg/mL. These results were interpreted as moderately active and similar to those reported by Paulo et al. (2010) and Vestergaard and Ingmer (2019). Variation in activity toward clinical isolates and ATCC strains can be attributed to virulent factors the isolate has, such as biofilm formation ability that can hinder the antimicrobial penetration through bacterial cell walls. In addition, the potency of resveratrol is not only attributed to antimicrobial activity, but also to the potent anti-QS activity, which was proven by a significant reduction of 80% of the transcript levels of the* lasB* gene. The obtained results were in agreement with many other reports, which proved that resveratrol has potential health benefits (Tomé-Carneiro et al. 2013). Finally, the results were also proved by an approximate total eradication of bacteria in the mice group treated with resveratrol. *P. aeruginosa*-infected wounds are usually accompanied by trophic ulceration and fungal infection, and require long-term treatment. Possessing high antifungal, moderate antimicrobial, and low side effects over long periods has made resveratrol one of the best choices for the treatment of that kind of wound (Vestergaard and Ingmer 2019; Shevelev et al. 2020).

Wound healing involves the collaboration of several biological and immunological factors. Numerous mechanisms are involved in this complicated process, including inflammation, proliferation, neovascularization, fibrous tissue accumulation, wound contraction, remodeling, and maturation (Bhaskar Rao et al. 2015). Sometimes, especially in infectious conditions, these healing processes are not enough to restore epithelial integrity (Ruffin and Brochiero 2019). It has been demonstrated that *P. aeruginosa* is a common opportunistic pathogen and nosocomial bacterium that can infect wounds and alter the repair mechanisms, resulting in persistent wounds. Prolonged antibiotic administration does not efficiently reduce the number of bacteria in a persistent wound due to the higher risk of allergies and the possibility of drug resistance in some bacterial infections (Wilson et al. 2005). Consequently, it is therapeutically necessary to find a topical therapeutic alternative that can reduce the high bacterial burden of the wound while accelerating the healing process. Therefore, one of the objectives of this study was to examine the role of the topical application of resveratrol in reducing bacterial load as well as enhancing the healing process in *P. aeruginosa*-infected wounds. In this investigation, histopathological examination of the control non-infected wound revealed normal healing processes. Conversely, the *P. aeruginosa*-infected wound revealed an obvious delay in the repair processes. This delay may be attributed to the contribution of bacterial infection in the cytokines' and growth factors’ degradation, resulting in the suppression of several cellular processes involved in the healing of wounds (Payne et al. 2003). These suggestions were confirmed by the current work findings of both immunohistochemical and molecular assays. The immunohistochemical analysis revealed that the expression of *αSMA* in the myofibroblasts was at the normal levels in control non-infected wounds, but weakly expressed in *P. aeruginosa*-infected wounds. *αSMA* has a plethora of actions in wound healing via acceleration of wound closure by the contractile force of myofibroblasts (Mazumdar et al. 2021). In open wounds, the dermal fibroblast begins to proliferate, migrate to the wound bed and edges, and differentiate into myofibroblasts by the action of *αSMA*.

The obtained results were confirmed by the downregulation of the mRNA levels of *VEGF* in the P. *aeruginosa*-infected group, indicating a delay in angiogenesis and subsequently delayed healing processes. *VEGF* is generated by activated macrophages, platelets, and keratinocytes. It plays a crucial role in the process of angiogenesis and granulation tissue formation by encouraging the growth of new blood vessels (Ferrara 2004). At the site of injury, the growth of new blood vessels is associated with increased *VEGF* transcription (Shim et al. 2013). The inflow of inflammatory cells into the site of damage, vascular permeability, and endothelial cell proliferation are all aspects of *VEGF*'s participation in wound healing (Galiano 2004), whereas significant upregulation of the mRNA levels of *TNF-α* and *IL-1β* was recorded in the same group, indicating an excessive inflammatory condition. The process of wound healing is orchestrated by various cells and signaling molecules (Tyavambiza et al. 2022). After an injury, neutrophils are the first cells to enter the wound site and release pro-inflammatory mediators like *TNF-α*, *IL-1β*, and *IL-6,* which attract additional neutrophils with other immune cells and intensify the inflammatory process (Ellis et al. 2018).

Recently, several studies have revealed that resveratrol has anti-inflammatory, antioxidant, angiogenic, and cell-protective properties (Şener et al. 2018). All these valuable effects make it useful for the acceleration of wound healing, scarring, and skin photoaging (Hecker et al. 2022). The repairing process of resveratrol was demonstrated through the improvement of the histopathological picture and increasing the immunoexpression of *αSMA* that exceeded those of the control uninfected wound. Additionally, our results revealed that resveratrol increased the mRNA levels of *VEGF*. Angiogenesis is essential for wound healing, and resveratrol may have an impact on neovascularization (Bilgic 2021). Numerous investigations revealed that resveratrol could stimulate the expression of *VEGF*, which in turn controls angiogenesis (Khalaf et al. 2019; Lakshmanan et al. 2019). According to Christovam et al. (2019), resveratrol activates the silent information regulator sirtuin 1 (SIRT1), which is associated with collagen organization, fibroplasia, and angiogenesis. Furthermore, our results showed that resveratrol decreased the mRNA levels of pro-inflammatory cytokines (*IL-1β* and *TNF-α*), in accordance with Ding et al. (2022) who found similar results in a murine diabetic wound model. Besides the immunostimulant potential of resveratrol at the site of the wound, it is also associated with antibacterial and antifungal activities (Zhai et al. 2015). In the context of non-healing wounds, Shevelev et al. (2020) demonstrated that topical resveratrol treatment exhibited a significant antimicrobial efficacy against *Candida albicans*, *Pseudomonas aeruginosa*, and *Staphylococcus aureus*, which are considered significant pathogens. The antibacterial properties of resveratrol appeared to be even more potent in comparison to other commercial antibacterial ointments (Konyalioglu et al. 2013). From the previously mentioned findings of our histopathological, immunohistochemical, and molecular analysis, it was evident that resveratrol treatment revealed an acceptable wound-healing effect besides its antibacterial properties.

## Conclusion

Resveratrol advances the healing process via the shortening of the inflammatory stage. This is achieved by reducing the expression of the pro-inflammatory cytokines in addition to stimulating cellular proliferation and angiogenesis in the proliferation phase via upregulating *αSMA* and *VEGF* expressions. Taken together, this study shows resveratrol to be a promising safe antivirulent and antimicrobial agent that can overcome and improve the healing of infected wounds caused by hypervirulent multidrug-resistant clinical *P. aeruginosa*. The strong association between the QS system, antimicrobial resistance, and virulence genes acts to overcome severe *P. aeruginosa* infections, especially in hospitalized patients under high antibiotic pressure. Therefore, a successful treatment protocol should cover those three aspects to ensure full eradication. This study supports resveratrol anti-*Pseudomonas aeruginosa* activity due to its high anti-QS, direct antibacterial activity, direct antifungal, and immune-stimulating action by increasing macrophages’ infiltration.

## Data Availability

All data generated or analyzed exist in the submitted manuscript.

## Abbreviations

CFU: Colony forming unit
ESKAPE: *Enterococcus faecium*, *Staphylococcus aureus*, *Klebsiella pneumoniae*, *Acinetobacter baumannii*, *Pseudomonas aeruginosa*, And *Enterobacter* species
*IL-1β*: Interleukin-1β
MBC: Minimum bactericidal concentration
MIC: Minimum inhibitory concentration
*TNF-α*: Tumor necrosis factor-α
*VEGF*: Vascular endothelial growth factors
*αSMA*: Alpha-smooth muscle actin
qRT-PCR: Quantitative real-time reverse-transcription PCR
